# 190 A feasibility randomised controlled trial of an intervention to increase physical activity and reduce sedentary behaviour in people with severe mental illness

**DOI:** 10.1093/eurpub/ckae114.034

**Published:** 2024-09-26

**Authors:** Sarah Howes, John Brady, Mary Clarke, Mike Clarke, Maurice Dillon, Judith McAuley, Catherine McDonough, Marie Murphy, Ailsa Niven, Mark Tully, Julie Williams, Iseult Wilson, Suzanne McDonough

**Affiliations:** Ulster University, United Kingdom; Health and Social Care Northern Ireland, United Kingdom; Health Service Executive, Ireland; Queen’s University Belfast, United Kingdom; Health Service Executive, Ireland; Health and Social Care Northern Ireland, United Kingdom; Health Service Executive, Ireland; Ulster University, United Kingdom; University of Edinburgh, United Kingdom; Ulster University, United Kingdom; King’s College London, United Kingdom; Queen’s University Belfast, United Kingdom; Royal College of Surgeons in Ireland, Ireland

## Abstract

**Purpose:**

People with severe mental illness (SMI), such as schizophrenia, psychosis, bipolar disorder and major depressive disorder, are less physically active and more sedentary than healthy controls, contributing to poorer physical health outcomes in this population. This study aimed to test the feasibility of a multi-component behaviour change intervention aimed at increasing physical activity (PA) and reducing sedentary behaviour (SB) compared with a one-off education session in people with SMI.

**Methods:**

The Walking fOR Health (WORtH) study was a 13-week feasibility randomised controlled trial (RCT) that recruited adults with SMI. Participants were randomised (2:1) to the WORtH intervention or a one-off education session. The WORtH intervention comprised an education session, a wrist-worn activity monitor and six health coaching sessions. Primary outcomes were feasibility (recruitment, retention and adherence rates) and acceptability (semi-structured interviews with participants and clinicians delivering the intervention). Secondary outcomes, including device-measured (Axivity AX3) and self-reported PA and SB, were reported descriptively.

**Results:**

Fifty-four participants (25 male:29 female; mean age 51.6 years) were recruited, representing 90% target recruitment, and 94% provided follow-up data. Adherence with all core intervention components was >80%. Qualitative feedback indicated high levels of satisfaction, particularly related to walking an acceptable form of PA and the activity tracker and coach. Valid device-measured moderate-vigorous PA (MVPA), the intended primary outcome for a definitive trial, was obtained from 90% participants at baseline and 75% participants at post-intervention. Point estimates indicated a mean increase of 8.6 minutes/day of MVPA in the intervention group at post-intervention, and a mean increase of 1.0 minute/day in the control group.

**Conclusions:**

The findings of this study support the feasibility of a multicomponent behaviour change intervention aimed at improving PA and SB in adults with SMI and will be used to optimise the design of a definitive RCT. Supporting adults with SMI to engage in and sustain PA could reduce the health disparity observed in this population.

**Support/Funding Source:**

Funding by the European Union’s INTERREG VA Programme, managed by the Special EU Programmes Body, awarded to the Health and Social Care Research & Development Division Cross-border Healthcare Intervention Trials in Ireland Network (CHITIN) programme.

